# Acknowledgement to Reviewers of the *Journal of Clinical Medicine* in 2014

**DOI:** 10.3390/jcm4010124

**Published:** 2015-01-08

**Authors:** Journal of Clinical Medicine Editorial Office

**Affiliations:** MDPI AG, Klybeckstrasse 64, CH-4057 Basel, Switzerland

The editors of the *Journal of Clinical Medicine* would like to express their sincere gratitude to the following reviewers for assessing manuscripts in 2014:

Al-Dujaili, Emad
Allolio, Bruno
Ambasudhan, Rajesh
Anand-Apte, Bela
Angel, Michael J.
Ashkenazi-Hoffnung, Liat
Baroni, Argelinda
Bello, Salvador
Benn, Peter
Bernal-Mizrachi, Carlos
Bezinover, D.
Bhandari, Pradeep
Bhat, Pangala V.
Biden, Trevor
Binah, Ofer
Blaner, William
Blurton-Jones, Mathew
Boscolo-Rizzo, Paolo
Bouza, Emilio
Bruce, Duff
Bühling, Frank
Burke, Michael
Califaretti, Nadia
Carey, Louise
Carlberg, Carsten
Carneiro, Ângela
Carr, Amanda
Carter, John A.
Charumathi, Sabanayagam
Chen, Gwo-Shing
Chen, Shu-Tzu
Chetty, Shilpa
Chmurzynska, Agata
Chun, Taehoon
Consiglio, Antonella
Couillard, Charles
Cowan, Morton J.
Creutzig, Ursula
Crouch, Rosalie K.
Cuckle, Howard
Dam, Gitte
Davidson, Kathryn C.
De Mast, Quirijn
De Peppo, Giuseppe Maria
De Sousa Lopes, Susana Chuva
Debenedetto, Anna
DessìAngelica, Angelica
Devin, Jessica K.
Dhar, Rajat
Dietrich, W. Dalton
Dilley, Rodney
Diringer, Michael N.
Donoso, Larry A.
Dushay, Jody
Dusso, Adriana
Ellis, James H.
Erceg, Slaven
Espinoza, Gabriela M.
Esteban, Andrés
Evans, Mark
Exadaktylos, Aristomenis
Ferrara, Felicetto
Ferro, Jose M.
Furukawa, Masayuki
Futamura, Masaki
Gaglio, Paul
Gailhouste, Luc
Galanter, Cathryn A.
Ghidini, Alessandro
Goetzl, Laura
Goldstein, Benjamin I.
Goossens, An
Gorin, Michael
Goureau, Olivier
Grattagliano, Ignazio
Greenberg, Arthur
Grisanti, Salvatore
Gritti, Paolo
Harper, Gordon
Hasegawa, Hajime
Hatton, Sean
Hattori, Fumiyuki
Henderson, Andrew
Herland, Anna
Hoffman, Martin
Holland, William L.
Holtan, Shernan
Hundal, Harinder S.
Issa, P. Charbel
Ito, Daisuke
Iwata, Takeshi
Izadyar, Fariborz
Jayasekera, Jinani
Jayasena, Channa
Johnston, Donna L.
Jong, Eiko de
Jung, Alain C.
Justo, Dan
Kaplan, Henry J.
Karp, Judith
Kasten, Mary J.
Katie Mahon, Katie
Katz, Lorraine E. Levitt
Kawamata, Shin
Kelly, Daniel F.
Kim, Keung Nyun
Kim, Sung-Hyun
Kleindienst, Andrea
Knechtle, Beat
Knoepfler, Paul S.
Knott, R.
Kota, Vamsi
Krantz, David
Kronvall, Erik
Kume, Shoen
Kwa, Faith A. A.
Kwak, Shin
Larsen, Jörg
Larsen, Michael
Laurie, Karen
Laville, Maurice
Lee, Kwang Hoon
Lee, Moon Soo
Lee, Yoon Kwang
LeRoith, Derek
Lestuzzi, Chiara
Levis, Hannah
Lin, Wen-Yuan
Lipton, Allan
Loeb, David M.
Ma, Hon Ming
Macedo, M. Paula
Maesaka, John K.
Marcucci, Fabrizio
Marcucci, Guido
Marigo, Valeria
Marik, Paul E.
Marin-Castaño, Maria E.
Meaney, Calvin J.
Mees, Sanne M. Dorhout
Melendy, Tom
Michelis, Michael F.
Mitterhofer, A.P.
Molina-Cabrillana, J.
Montagnese, Sara
Moore, Elizabeth
Moura, Ivan Cruz
Murphy, Theresa
Naito, Yuji
Nakahara, Tomomi
Nakatsuka, Ryusuke
Navasa, Miquel
Negrea, Lavinia
Nestler, U.
Nguyen, Minh Ly
Nishikawa, Keizo
Nonoyama, Shigeaki
Nussenblatt, Robert B.
OBrien, Louise
O'Callaghan, Anna R.
Odibo, Anthony
Oishi, Akio
Paludan, Søren R.
Pano-Pardo, Jose Ramon
Pasquini, Marcelo C.
Patruno, Vincenzo
Pergament, Eugene
Pham, Phuong-Chi T.
Phillips, Mary L.
Polo, Jose
Poo, Haryoung
Rachmilewitz, Eliezer A.
Radeke, Monte J.
Radovini, Sandra
Rassaf, Tienush
Ray, Udayan
Reid, Lola M.
Rhoney, Denise H.
Rincón, Olga Gómez del
Rodrigo, Chamira
Rondon-Berrios, Helbert
Rose, Peter S.
Rosendeld, Philip
Rosenzweig, Steven A.
Roth, Daniel E.
Rou, CW le
Runkle, I.
Sakurai, Hidetoshi
Saslow, Debbie
Schuster, Inge
Schweikart, Karen
Selvaraj, Vimal
Sharma, Vijay K.
Sigal, Samuel H.
Singh, Manpreet
Soiza, Roy L.
Sparrow, Janet R.
Sridharan, Shankar
Staton, Dennis
Stewart, Michael W.
Stoddard, Joel
Storm, Tina
Su, Emily
Sugaya, Makoto
Sundar, Krishna M.
Surti, Urvashi
Szeszko, Philip R.
Tarantino, Giovanni
Tauro, Sudhir
Teti, Diana
Thomas, Helen E.
Thomas, Jaiyeola
Thomsen, Henrik S.
Tien, Hwei-Fang
Tigizov, Sharof M.
Tomishima, Mark
Torrone, Elizabeth
Tung, Leslie
Unick, Jessica L.
Vantyghem, Marie-Christine
Vavvas, Demetrios G.
Veraldi, Stefano
Wang, Aijun
Washburn, Jason
Welm, Alana
White, Helen
Wolf, Dominik
Wolitski, Richard
Woods, Niels-Bjarne
Wu, Huaizhu
Xu, Chunhui
Xu, Xiao-ming
Xu, Yiqing
Yang, Paul
Yee, Jiing-Kuan
Yessayan, Lenar
Yoshihara, Satoshi
Zhang, Yong
Ziemssen, Focke
Zimmerlin, Ludovic
Zimmerman, Jerry J.
Zipfel, Peter

